# Smartphone-based prediction of dopaminergic deficit in prodromal and manifest Parkinson’s disease

**DOI:** 10.1038/s41746-025-02148-2

**Published:** 2025-12-01

**Authors:** Katarina M. Gunter, Karolien Groenewald, Timothee Aubourg, Christine Lo, Jessica Welch, Jamil Razzaque, Ludo van Hillegondsberg, Adriana Nastasa, Pietro-Luca Ratti, Beatrice Orso, Pietro Mattioli, Matteo Pardini, Stefano Raffa, Federico Massa, Daniel R. McGowan, Kevin M. Bradley, Dario Arnaldi, Johannes C. Klein, Siddharth Arora, Michele T. Hu

**Affiliations:** 1 Nuffield Department of Clinical Neurosciences, University of Oxford, Oxford, UK; 2 Department of Clinical Neurology, Sheffield Teaching Hospitals NHS Foundation Trust, Sheffield, UK; 3 Department of Neuroscience, University of Genoa, Genoa, Italy; 4 Clinical Neurophysiology, IRCCS Ospedale Policlinico San Martino, Genoa, Italy; 5 Clinical Neurology, IRCCS Ospedale Policlinico San Martino, Genoa, Italy; 6 Nuclear Medicine Unit, IRCCS Ospedale Policlinico San Martino, Genoa, Italy; 7 Department of Medical Physics and Clinical Engineering, Oxford University Hospitals NHS Foundation Trust, Oxford, UK; 8 Department of Oncology, University of Oxford, Oxford, UK; 9 Department of Nuclear Medicine, Oxford University Hospitals NHS Foundation Trust, Oxford, UK; 10 Wales Research and Diagnostic PET Imaging Centre, Cardiff University, Cardiff, UK; 11 Somerville College, University of Oxford, Oxford, UK; 12 Saïd Business School, University of Oxford, Oxford, UK

**Keywords:** Parkinson's disease, Brain imaging

## Abstract

Dopamine transporter (DaT) SPECT can confirm dopaminergic deficiency in Parkinson’s disease (PD) but remains costly and inaccessible. We investigated whether brief smartphone-based motor assessments could predict DaT scan results as a scalable alternative. Data from Oxford and Genoa cohorts included individuals with iRBD, PD, and controls. Machine learning models trained on smartphone-derived features classified DaT scan status and predicted striatal binding ratios, compared with MDS-UPDRS-III benchmarks. Among 100 DaT scans, the smartphone-only XGBoost model achieved AUC = 0.80, improving to 0.82 when combined with MDS-UPDRS-III (AUC’s gender-corrected). A simpler logistic regression model performed better with MDS-UPDRS-III alone (AUC = 0.83) versus smartphone features, with slightly higher performance when combined (AUC = 0.85). Regression models predicted binding ratios with modest error (RMSE = 0.49, R² = 0.56). Gait, tremor, and dexterity features were most predictive. These findings support smartphone-based assessments complementing clinical evaluations, though larger independent validation remains essential.

## Introduction

Parkinson’s disease (PD) is a progressive neurodegenerative disorder primarily characterised by the loss of dopaminergic neurons in the nigrostriatal pathway, leading to hallmark motor symptoms such as tremor, bradykinesia, and rigidity. Dopamine transporter (DaT) single-photon emission computed tomography (SPECT) imaging is commonly used to visualise and quantify dopaminergic function in the striatum. It plays an important role in clinical diagnostics by distinguishing PD from non-degenerative mimics such as essential tremor and is increasingly used as an inclusion criterion in disease-modifying clinical trials^1^.

A key metric derived from DaT SPECT imaging is the striatal binding ratio (SBR)—a semi-quantitative measure of dopamine transporter availability in key regions of interest (ROIs), including the caudate and putamen. This quantitative measure has been shown to correlate with and predict various aspects of disease progression, including motor dysfunction^1,2^. Several studies have demonstrated a significant inverse relationship between contralateral SBR values and Movement Disorder Society–Unified PD Rating Scale Part III (MDS-UPDRS-III) motor scores in PD, with lower SBR values indicating greater dopaminergic loss and worse motor function. Over a four-year period, Yang et al. reported a significant association (p = 0.037) between the SBR and MDS-UPDRS-III scores^3^. Similarly, Kerstens et al. identified a significant (p < 0.04) negative correlation between the MDS-UPDRS-III and striatal binding in PD patients who were off levodopa^4^. Interestingly, strongest inverse correlations between contralateral striatal binding were found with motor symptoms of bradykinesia, posture, gait and other midline symptoms including, speech and facial expression, rather than rigidity^5^. Despite this clinical utility, DaT imaging remains costly, requires specialised equipment and incurs exposure to ionising radiation, limiting frequent use.

The current lack of any disease modifying treatment for PD has led to increasing interest in prodromal forms that might offer the opportunity to intervene earlier in the disease course. iRBD represents one of the most well-characterised prodromal markers of alpha-synucleinopathies converting to PD and Dementia with Lewy Bodies (DLB). It is associated with a 6% annual risk of phenoconversion to PD/DLB^6^, with post-mortem neuropathology showing that alpha-synuclein was the predominant driving pathology in all 20 cases^7^. One study has shown that over 60% of individuals with iRBD exhibit early nigrostriatal dysfunction on DaT SPECT or transcranial ultrasound imaging^8^. 30% of these subjects went on to develop an alpha-synucleopathy after a period of 2.5 years. Furthermore, the Parkinson’s At Risk Study showed that patients with anosmia and other prodromal PD symptoms including iRBD exhibit alterations in DaT SPECT^9^. Identifying dopaminergic deficits in iRBD could therefore support targeted recruitment and trial enrichment strategies for prodromal PD. However, while literature suggests that DaT SPECT – if properly semi-quantified – can be used at a single subject level in prodromal PD^10^, clear cut-off values to stage patients across the whole prodromal to overt PD stage continuum are still missing. These constraints motivate scalable digital assessments.

Digital health tools offer a practical route to wider screening. Previous studies have demonstrated that the 8-minute Oxford Parkinson’s Disease Centre (OPDC) smartphone application can differentiate between healthy controls (HCs), individuals with isolated REM sleep behaviour disorder (iRBD), and PD participants, achieving pairwise sensitivities and specificities between 84.6 and 91.9%^11^. The application has also shown some promise in predicting the MDS-UPDRS-III motor scores—a standardised clinical scale used to quantify motor symptom severity in PD, where higher scores indicate greater impairment^12^. Individuals with iRBD are not typically evaluated using the MDS-UPDRS-III in clinical practice, despite evidence indicating the presence of motor symptoms prior to phenoconversion^13^. However, several longitudinal iRBD cohort studies have shown that gradually increasing MDS-UPDRS III scores approaching those seen in overt PD occur in the 5 years prior to phenoconversion^6,14^. Furthermore, we have demonstrated that a single smartphone test can accurately predict meaningful clinical transition points for people with Parkinson’s including the onset of gait freezing, falls and cognitive impairment 18 months prior to onset^15^.

However, few studies have focused on predicting DaT scan results, which are inherently resource-intensive assessments, and do not address the limitations of cost and availability^16^. Based on the established relationship between motor impairment and DaT binding, this study investigates whether smartphone-derived motor features can predict DaT scan abnormalities and striatal binding ratios. Importantly, in this work, “prediction” refers to the ability to characterise current dopaminergic status—specifically, whether a participant has a normal or abnormal scan, and the extent of dopaminergic loss as measured by striatal binding ratio—rather than forecasting future clinical progression.

Such a digital framework could significantly reduce costs, expand accessibility, and facilitate screening in larger prodromal and early PD populations—including those with iRBD—who could benefit from early detection of dopaminergic deficits. This study investigates whether smartphone-derived motor features can predict DaT scan abnormalities and SBR. Leveraging smartphone-based tools to stratify individuals by their likelihood (or probability) of abnormal DaT scans may help quantify individual phenoconversion risk in clinical and research settings, particularly when combined with easy to measure clinical predictors. By predicting DaT binding ratios, we aim to objectively measure the extent of dopaminergic impairment, laying the groundwork for a digital biomarker that could be used in disease-modifying trials.

## Results

### Participant data

**Fig. 1:**
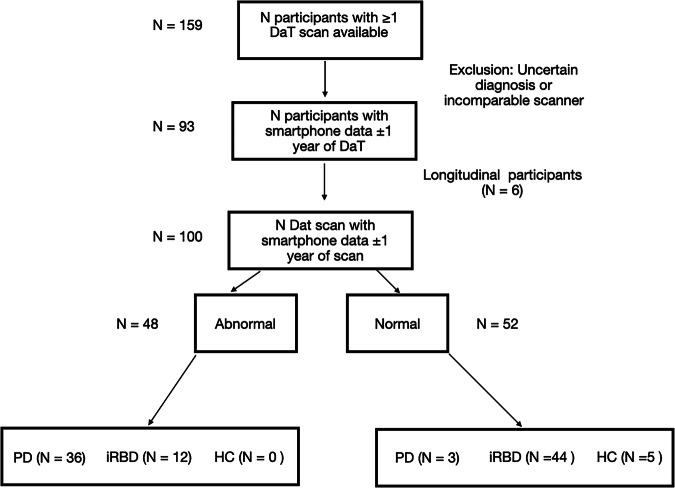
Flow diagram of participant and DaT SPECT inclusion. Parkinson’s disease (PD), isolated REM-sleep-behaviour disorder (RBD), and healthy controls (HC).

Of the participants who completed both smartphone assessments and DaT scans, 93 (5 HCs, 49 iRBD, and 39 PD) had assessments that fell within ±1 year of the corresponding scan. This one-year interval was chosen by consensus among three imaging neuroscientists, assuming no substantial change in SBR would occur within that timeframe. Sixty-eight of these participants were from the OPDC Discovery cohort, while 25 were from the Genoa cohort. Six participants had longitudinal DaT scans matched to longitudinal smartphone assessments, yielding a total of 100 unique DaT scans. Of these, 52 were classified as normal and 48 as abnormal. Table 1 summarises the demographics of this sample, and a flow diagram is given in Fig. 1. The MDS-UPDRS-III was significantly different between the two groups (p = <0.00001). Although both groups were predominantly male, the sex distribution differed significantly between normal and abnormal DaT scan groups, with females showing a higher proportion of abnormal scans (p = 0.00001).

**Table 1:** Demographic and clinical characteristics of participants with normal vs abnormal DaT Scans

	Normal DaT Scan	Abnormal DaT Scan	P-value
N participants^a^	45	48	-
N DaT Scans	48	52	-
N DaT Scans PD/RBD/HC	3/44/5	36/12/0	-
N smartphone recordings/participant (μ ± SD)	12.08 ± 14.67	6.75 ± 8.69	-
Sex (male %)	84.14	72.22	**0.00001**
Age (μ ± SD)	67.56 ± 8.02	68.32 ± 8.72	0.20
MDS-UPDRS-III (μ ± SD)	5.52 ± 6.24	25.69 ± 11.82	**<0.00001**
Interval between recording and scan (absolute days)	191 ± 147	183 ± 137	0.41
Interval (recording pre scan)	194 ± 165	165 ± 145	**0.04**
Interval (recording post scan)	189 ± 129	206 ± 121	0.06

Values are presented as mean ± standard deviation or percentages, with p-values indicating differences between the two groups. The interval values represent the time (in days) between smartphone recording and DaT scan. Bold values indicates statistical significant *P* values (*p* < 0.05).

^a^For participants with longitudinal scans, abnormal versus normal classification was based on the status at their first available DaT scan.

Table 2 presents the binding ratio statistics for the four striatal ROIs overall and by diagnosis group, with further details provided in Supplementary Tables 1 and 2. In the right caudate, PD participants had a significantly lower SBR than HCs (mean difference = −1.11; 95% CI, −1.24 to −0.99; p < 0.001) and RBD (mean difference = 0.80; 95% CI, 0.72 to 0.87; p < 0.001), while RBD also showed a lower SBR than HCs (mean difference = −0.32; 95% CI, −0.43 to −0.20; p < 0.001). Similar significant differences were observed in the left caudate and both putamen. In RBD, the left caudate was significantly lower than both putamen regions, with additional significant differences noted between the left putamen and right caudate, as well as between the right caudate and right putamen. No differences were found between the left and right caudate or between the left and right putamen. A comparable pattern was observed in PD, except for the lack of differences between the left vs. right caudate and putamen. Among HCs, there were no significant pairwise differences, indicating uniform binding across regions.

**Table 2:** Mean and standard deviation of binding ratios in each region of interest

	Right Putamen (μ ± SD)	Left Putamen (μ ± SD)	Right Caudate (μ ± SD)	Left Caudate (μ ± SD)	Putamen Asym^a^ (μ ± SD)	Caudate Asym^a^ (μ ± SD)
All Ratio	3.10 ± 0.74	3.21 ± 0.79	3.57 ± 0.61	3.56 ± 0.63	−0.11 ± 0.40	0.01 ± 0.37
HC Ratio	3.88 ± 0.36	3.89 ± 0.37	4.11 ± 0.57	4.06 ± 0.40	−0.01 ± 0.25	0.06 ± 0.30
RBD Ratio	3.44 ± 0.47	3.54 ± 0.57	3.79 ± 0.46	3.75 ± 0.47	−0.10 ± 0.32	0.04 ± 0.30
PD Ratio	2.24 ± 0.35	2.38 ± 0.54	3.00 ± 0.39	3.07 ± 0.64	−0.15 ± 0.54	−0.07 ± 0.48

Overall and within each diagnosis group; healthy control (HC), REM-sleep-behaviour disorder (RBD), and Parkinson’s disease (PD).

^a^Asymmetry given as right - left hemisphere.

### Predicting abnormal DaT scans

**Fig. 2:**
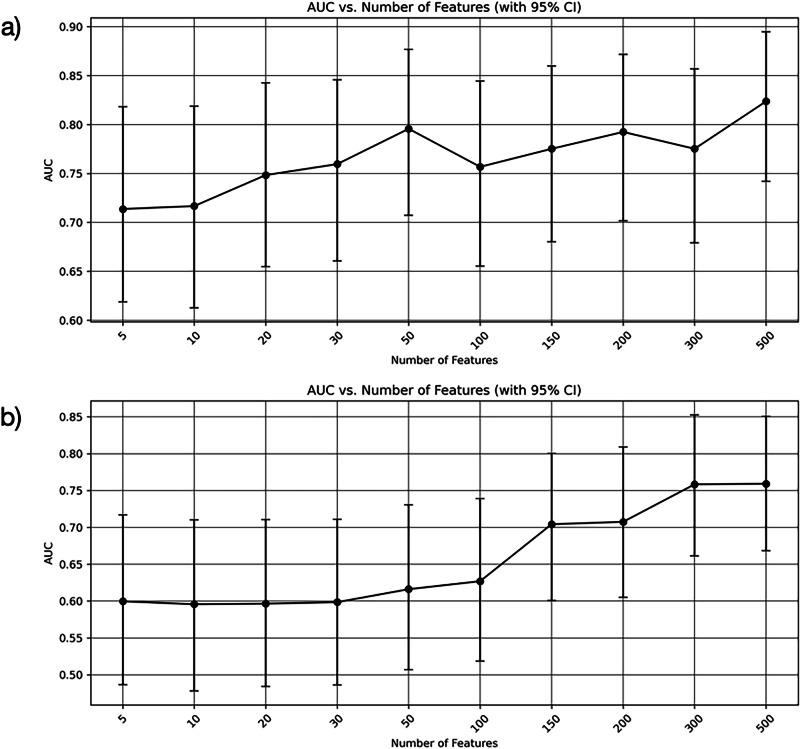
Mean Area-Under-the-Curve (AUC) for varying number of smartphone features. Mean AUC with 95% confidence intervals (CI) across 5-fold cross-validation for varying number of smartphone features using **a** the XGBoost model, and **b** the logistic regression model.

We next evaluated whether smartphone features, alone or combined with MDS-UPDRS-III, could classify DaT status across this two-centre sample. Table 3 and Table 4 show the performance of XGBoost and logistic regression (LR) models for predicting normal vs abnormal DaT scans using: (1) only the MDS-UPDRS-III, (2) only the smartphone features, and (3) both MDS-UPDRS-III and smartphone features. The mean AUC across the 5-folds with the 95% CIs for the smartphone-only models using varying number of features are shown in Fig. 2. The best performing XGBoost smartphone model (500 features) achieved an AUC comparable to the model using the in-clinic MDS-UPDRS-III score only (AUC: 0.82 and 0.81, respectively). When combining the MDS-UPDRS-III with the top 500 (out of a total of 1057) smartphone features, the model achieved the best performance, with an AUC of 0.84 (95% CI: 0.75 to 0.92) and balanced accuracy of 0.84. The best performing LR smartphone model (500 features) did not achieve as high an AUC as using the LR MDS-UPDRS-III only model (AUC 0.76 and 0.88, respectively). However, the combined smartphone and MDS-UPDRS-III LR model achieved an AUC of 0.88 with an elevated confidence interval range compared to the MDS-UPDRS-III only model. To assess the robustness of the classification models, we performed repeated cross-validation across multiple random splits of the data. The mean performance metrics along with their standard deviations are reported in Supplementary Table 3 and 4 for the XGBoost and LR models, respectively. Confusion matrices show the aggregated classifications for each of the diagnosis subgroups in Supplementary Fig. 1 (combined smartphone features + MDS-UPDRS-III models). The LR model achieved a higher classification performance in RBD participants, whereas the XGBoost performed better in patients with PD.

**Table 3:** Performance of each of the three XGBoost classification models

	AUC ± SD (95% CI)	AUC adjusted ± SD (95% CI)	Sensitivity ± SD	Specificity ± SD	Balanced accuracy ± SD (Sensitivity + Specificity)/2
MDS-UPDRS-III	0.85 ± 0.04(0.72–0.90)	0.79 ± 0.05(0.68-0.88)	0.68 ± 0.23	0.89 ± 0.10	0.79 ± 0.13
Smartphone features (XGBoost)	0.84 ± 0.11 (0.74–0.90)	0.80 ± 0.05 (0.72–0.88)	0.68 ± 0.11	0.80 ± 0.19	0.74 ± 0.11
Smartphone features + MDS-UPDRS-III (XGBoost)	0.88 ± 0.05 (0.75-0.92)	0.82 ± 0.05 (0.72–0.90)	0.76 ± 0.10	0.91 ± 0.11	0.84 ± 0.07

The AUC adjusted for the effect of sex is also reported (AUC adjusted). Balanced accuracy is the average of sensitivity and specificity. SD: standard deviation of the error metric.

Reported using the Area-Under-the-Curve (AUC) with 95% confidence intervals (CI), sensitivity, and specificity.

**Table 4:** Performance of each of the three logistic regression (LR) classification models

	AUC ± SD (95% CI)	AUC adjusted ± SD (95% CI)	Sensitivity ± SD	Specificity ± SD	Balanced accuracy ± SD (Sensitivity + Specificity)/2
MDS-UPDRS-III (LR)	0.88 ± 0.02 (0.78 - 0.94)	0.83 ± 0.04 (0.76-0.92)	0.70 ± 0.15	0.93 ± 0.09	0.82 ± 0.08
Smartphone features (LR)	0.76 ± 0.09 (0.66-0.85)	0.73 ± 0.05 (0.64-0.83)	0.73 ± 0.09	0.75 ± 0.16	0.74 ± 0.09
Smartphone features + MDS-UPDRS-III (LR)	0.88 ± 0.04 (0.80-0.94)	0.85 ± 0.04 (0.77-0.93)	0.79 ± 0.08	0.89 ± 0.10	0.84 ± 0.06

Reported using the Area-Under-the-Curve (AUC) with 95% confidence intervals (CI), sensitivity, and specificity. The AUC adjusted for the effect of sex is also reported (AUC adjusted). Balanced accuracy is the average of sensitivity and specificity. SD: standard deviation of the error metric.

A multiple linear regression model was applied to the output of the classification models to explore the effects of age and sex. Sex was a significant variable, with a coefficient of 0.16 (p = <0.00001) in the best performing XGBoost model (Supplementary Table 5) and 0.14 (p = <0.00001) for the best performing LR model. The probability outputs from the model were adjusted for sex, and the resulting adjusted AUC for the best performing XGBoost and LR models were 0.82 (95% CI: 0.72 to 0.90) and 0.85 (95% CI: 0.77 to 0.93), respectively (Table 3 and Table 4). The combined smartphone and MDS-UPDRS-III models outperformed the MDS-UPDRS-III only models with respect to the adjusted AUC.

SHapley Additive exPlanations (SHAP^17^) values were aggregated across all 5 CV folds to give an overall view of feature importance. Here we present the SHAP values for the XGBoost smartphone model, which outperformed its LR counterpart. The aggregated SHAP values for all observations are shown in Supplementary Fig. 2. Gait, rest-tremor, and voice were in the top 5 ranked smartphone features of significance in the model. The mean absolute SHAP values with standard deviation across the 5-folds are shown in Supplementary Fig. 3.

### Pre-screening sensitivity analysis

Because a practical use is triaging who to image, we tested performance in a milder cohort. To simulate this pre-screening use case, we retrained and evaluated the LR models after excluding participants with moderate to advanced PD, defined as having an MDS-UPDRS-III score of 33 or higher^18^. This led to the exclusion of six PD DaT scans. Following this adjustment, overall performance of all LR models declined, with the MDS-UPDRS-III–only model outperforming the others (see Table 5). Notably, the combined smartphone and MDS-UPDRS-III model showed a slightly lower standard deviation in AUC across folds.

**Table 5:** Performance of each of the three logistic regression (LR) classification models after removal of participants with moderate to severe Parkinson’s disease as defined by an MDS-UPDRS-III score of 33 or more

	AUC ± SD (95% CI)	Sensitivity ± SD	Specificity ± SD	Balanced accuracy ± SD (Sensitivity + Specificity)/2
MDS-UPDRS-III (LR)	0.83 ± 0.12 (0.74–0.92)	0.79 ± 0.10	0.84 ± 0.15	0.82 ± 0.09
Smartphone features (LR)	0.70 ± 0.08 (0.61–0.82)	0.60 ± 0.13	0.67 ± 0.09	0.64 ± 0.08
Smartphone features + MDS-UPDRS-III (LR)	0.80 ± 0.08 (0.72–0.90)	0.79 ± 0.08	0.89 ± 0.10	0.84 ± 0.06

Reported using the Area-Under-the-Curve (AUC) with 95% confidence intervals (CI), sensitivity, and specificity. The AUC adjusted for the effect of sex is also reported (AUC adjusted). Balanced accuracy is the average of sensitivity and specificity.

*SD* standard deviation of the error metric.

### Predicting striatal binding ratios

Finally, to test whether digital signals relate to quantitative dopaminergic loss, we modelled SBRs by region of interest (ROI). The performance of each ROI XGBoost regressor model is shown in Table 6. The best performing smartphone model used the top 300 smartphone features. The difference from the naïve benchmark was greatest in the right putamen. For most regions, the smartphone model had a slightly higher error than MDS-UPDRS-III alone but combining both resulted in the lowest error (RMSE = 0.49). The agreement between the actual and predicted right putamen values is shown in Supplementary Fig. 4 (R² = 0.56). A Bland-Altman plot demonstrated that the smartphone-only XGBoost model tended to under-predict higher striatal binding ratios, suggesting it was better at estimating values near the median. This bias may be partly attributable to the dataset’s limited size and imbalance, which could constrain the model’s ability to capture the full range of dopaminergic variability. However, the prediction plot for the combined XGBoost model showed the narrowest limits of agreement and no systematic error across the range (Supplementary Fig. 5). The decision tree regressor combining the smartphone features and the MDS-UPDRS-III did not perform as well as the XGBoost regressor. However, using the MDS-UPDRS-III alone in this model architecture achieved a lower RMSE compared to the XGBoost MDS-UPDRS-III model. The results for the decision tree models are summarised in Supplementary Table 6. For the right putamen, right caudate, and left caudate, combining the predictions from the MDS-UPDRS-III model and the smartphone model resulted in a lower RMSE compared to combining all features in the decision tree. This was not the case for the XGBoost model. To further examine the value of the addition of smartphone features, the smartphone features were fed into an XGBoost model to predict the residuals from the MDS-UPDRS-III DT model. The XGBoost model outperformed a naïve benchmark using the mean of the in-sample residuals as the prediction, with RMSE values of 0.50 and 0.56, respectively. The summary of these results can be found in Supplementary Table 7.

**Table 6:** Regression results for predicting the DaT ratios corresponding to the four regions of interest using the XGBoost models

Model	Right Putamen	Left Putamen	Right Caudate	Left Caudate
Naïve Benchmark	RMSE: 0.74 ± 0.42	RMSE: 0.79 ± 0.47	RMSE: 0.60 ± 0.37	RMSE: 0.65 ± 0.43
MDS-UPDRS-III	RMSE: 0.57 ± 0.38	RMSE: 0.66 ± 0.39	RMSE: 0.55 ± 0.33	RMSE: 0.61 ± 0.39
Smartphone features	RMSE: 0.63 ± 0.33	RMSE: 0.67 ± 0.38	RMSE: 0.63 ± 0.37	RMSE: 0.59 ± 0.36
Smartphone features + MDS-UPDRS-III	**RMSE: 0.49** ± **0.30**	**RMSE: 0.57** ± **0.34**	**RMSE: 0.52** ± **0.31**	**RMSE: 0.55** ± **0.37**
Combination prediction (mean)	RMSE: 0.64 ± 0.37	RMSE: 0.69 ± 0.39	RMSE: 0.57 ± 0.34	RMSE: 0.60 ± 0.35

RMSE: root mean squared error, presented as mean ± standard deviation.

The naïve benchmark issues the mean of the training data as a prediction for the entire test data. This benchmark serves as a reference point against which more sophisticated machine learning methods can be compared. Note: lower RMSE values are better. The best performing model is highlighted in bold.

### Correlation results

**Fig. 3:**
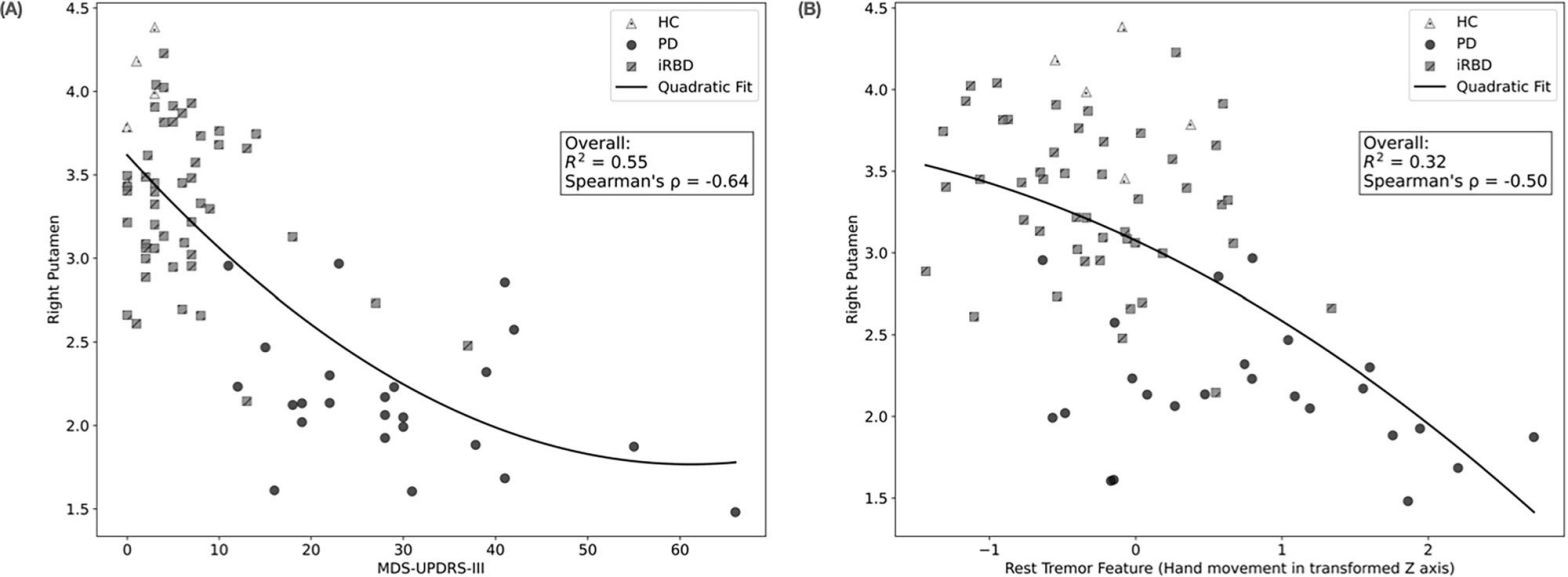
Scatter plots showing quadratic fits for right putamen binding ratio against two predictors. **A** MDS-UPDRS-III, and **B** a representative rest tremor feature (Entropy *Z*-axis), whereby a higher feature value corresponds to worse tremor symptoms. Each subplot includes data points from healthy controls (HC), RBD, and PD participants, along with the overall coefficient of determination (R^2^) and Spearman’s correlation coefficient, ρ.

The correlation between the MDS-UPDRS-III and the ROI was analysed (Supplementary Fig. 6). In the right putamen, we observed a Spearman’s coefficient of −0.64 and an R^2^ of 0.54 with a quadratic fit (Fig. 3A). The top smartphone features for ratio prediction (as determined by SHAP) from a random CV fold were also examined (Supplementary Fig. 7), with rest tremor features comprising most of the top 10. One of these features showed a Spearman’s coefficient of −0.50 and an R^2^ of 0.32 with the right putamen binding ratio (Fig. 3B).

## Discussion

Our previous work demonstrated that smartphone-based assessments can distinguish between disease groups and predict clinical motor scores such as the MDS-UPDRS-III^11^. This study demonstrates the feasibility of using smartphone-based motor assessments in combination with the MDS-UPDRS-III to predict DaT scan abnormalities in individuals with iRBD and PD. While the smartphone assessment is designed to approximate in-clinic motor evaluations, it captures features that differ from those a clinician may directly observe, offering finer resolution. We hypothesised that this added granularity, in addition to the gold-standard clinical motor measure, would be especially valuable for addressing the adjacent task of predicting dopaminergic deficit.

The main finding is that smartphone-only models performed comparably to MDS-UPDRS-III, and that combining the two improved discrimination. For classification, the combined XGBoost model reached AUC = 0.88, and results were robust after sex adjustment, indicating that motor features alone are strong predictors of dopaminergic deficiency. After post-hoc adjustments, the combined LR model also had a slightly higher performance compared to the MDS-UPDRS-III alone, with an AUC of 0.85 and 0.83, respectively. The simpler LR model outperformed the more complex XGBoost model when using only the MDS-UPDRS-III, with an AUC of 0.83 compared to 0.80 – reflecting the linear structure and low dimensionality of the clinical measure. This underscores the added value of integrating digital assessments while highlighting the importance of model selection based on data complexity and dimensionality.

We examined a pre-screening scenario by excluding moderate-advanced PD and re-evaluating the LR model. Following this adjustment, overall performance of all LR models declined, with the MDS-UPDRS-III–only model outperforming the others (see Table 5), while the combined model showed slightly lower variance across folds. These results suggest that the predictive value of motor assessments—particularly those derived from smartphone data—may be more limited in individuals with only subtle or early-stage motor signs. In this lower-severity cohort, clinical assessments appeared to retain stronger discriminative power, though the slightly reduced variance observed in the combined model may point to improved robustness. However, it is important to note that this analysis involved the removal of samples from an already small dataset, which may limit the reliability and generalisability of the observed trends.

Moving from a binary decision to quantification we explored whether the same signals track age-adjusted SBRs. While the smartphone-based XGBoost regressor showed slightly higher error than the MDS-UPDRS-III model for most regions, combining both data sources consistently reduced RMSE, particularly in the right putamen (RMSE = 0.49), where agreement between predicted and actual values was strongest (R² = 0.56; Supplementary Fig. 4). These results suggest that smartphone-based assessments can capture complementary motor information relevant to dopaminergic function. However, these gains were modest, and performance remained below what might be considered clinically actionable thresholds. Simpler models, such as decision trees, performed worse overall, and combining all features in a single decision tree often diluted performance. Interestingly, the MDS-UPDRS-III–only model performed better in the decision tree architecture than in XGBoost, which is expected, given that in low-dimensional settings, more complex models may not always offer advantages.

These quantitative findings align with prior imaging literature on motor function and dopaminergic loss. Earlier studies show that motor function correlates with striatal dopaminergic deficits in PD^19–21^. In one investigation, a significant negative correlation was found between UPDRS III and putamen binding in 27 PD participants^22^. Additional research has associated dopamine deficiency in the putamen with motor dysfunction, while caudate loss appears more closely tied to cognitive impairment in later disease stages^23,24^. Moreover, dopaminergic reduction in the putamen has also been observed in iRBD patients compared to controls^24–26^. Another study reported that adding DaT measures to clinical variables significantly improved the prediction of phenoconversion in iRBD^27^, underscoring the potential for digital assessments to be combined with other markers for identifying at-risk prodromal populations. Taken together with previous findings, our results reinforce the association between motor function and striatal dopaminergic loss, particularly in the putamen.

Finally, although smartphone features improved residual prediction over a naïve benchmark, the improvements were incremental. Plotting the predictions versus the actual values indicated that the smartphone only XGBoost model had bias towards under prediction for higher striatal binding ratios, potentially caused by the imbalance in the dataset (Supplementary Figs. 4 and 5). These findings point to the potential of smartphone-based assessments in supplementing clinical assessments for this task but also emphasise the need for further validation in larger, independent cohorts and the importance of understanding when and how different modelling approaches extract meaningful signal from noisy, real-world data.

Feature interpretation aligned with known motor–dopamine links. For DaT status classification, the most influential smartphone features were gait, rest tremor, voice, and dexterity; for SBR regression, balance also emerged. Prior work has revealed a significant inverse relationship between bradykinesia and striatal binding ratios^22,28^, which may be captured by the smartphone-based dexterity task metrics. One study examining UPDRS III and DaT binding ratios found inverse correlations between the normalised ratio and speech (*r* = −0.61), rigidity (*r* = −0.42), bradykinesia (*r* = −0.52), and posture/gait (*r* = −0.63) in 59 PD patients (p < 0.01)^29^, but no significant correlation with rest or action tremor—consistent with more recent work^30^. By contrast, our results indicate that smartphone-derived rest tremor features show a correlation with the right putamen at levels comparable to the correlation between MDS-UPDRS-III and the same region.

Compared to previous approaches, the high-frequency sampling and multiple dimensions of measurement (3-axis accelerometery) likely provide greater sensitivity to subclinical tremor manifestations that correlate with early dopaminergic deficiency. These findings highlight how granular digital features can rival a composite clinical score and may be sensitive to more subtle tremor manifestations. Given this, other sensors, including wearables, may also offer promising results for this task. Lonini et al. found that a single hand-worn sensor was sufficient to reliably detect bradykinesia and tremor in PD^31^. However, a systematic review of digital monitoring devices in PD, found that only 9 out of 73 devices were “recommended”, having strong correlation with established clinical metrics of motor function^32^. Notably, these were branded devices, such as Axivity, which require dedicated hardware. In contrast, our approach leverages consumer-grade smartphones – devices already widely owned by users – to perform active tasks like tapping and voice recording, enabling multimodal motor assessment without additional equipment. While current performance may vary slightly depending on smartphone model, future work will focus on cross-device validation to improve generalisability and scalability.

A key strength of this study is the rigorous evaluation of DaT scan abnormality by consensus among three independent experts. Including HCs, iRBD, and PD (100 unique DaT scans) broadens generalisability across dopaminergic deficiency. However, the small sample size may limit applicability to larger, more diverse populations. The use of only the MDS-UPDRS-III may also have constrained the models’ predictive performance. Since prodromal patients may not exhibit significant motor impairment, further work could focus on including other clinical metrics of motor function and non-motor function. Additionally, due to the presence of multiple versions of the smartphone app, only dominant hand features were used for bilateral tasks. Using features from the hand contralateral to the most affected side may offer more informative signals and could enhance model performance. With access to a larger dataset, we aim to explore the impact of additional factors—such as genetic information—by stratifying participants based on these variables to assess their influence on dopaminergic status and model performance.

In conclusion, this study demonstrates that motor features—captured through both clinical assessments and a smartphone-based application—can predict striatal dopaminergic deficits with accuracy comparable to in-clinic evaluation alone. By detecting subtle motor abnormalities remotely, the smartphone assessment offers a scalable and accessible complement to traditional clinical tools. When combined, the two approaches improved model performance, highlighting the value of integrating digital and clinical measures. Future approaches could combine simple home-based quantitative motor testing, for example the three metre time up and go (TUG) with the smartphone app to to identify prodromal and overt PD individuals with a higher likelihood of dopaminergic deficits. This would (i) aid triage of new referral pathways for suspected PD, improving the likelihood of earlier diagnosis and (ii) aid clinical trial selection, which increasingly stipulates dopaminergic deficit for study inclusion.

These findings carry important implications for early detection and ongoing monitoring in both prodromal and manifest PD. However, it is important to note that currently smartphone-based assessments may serve as a complementary, though not yet standalone, tool for detecting dopaminergic changes in both PD and at-risk populations. Further validation in larger and more diverse cohorts is needed to assess generalisability, clinical utility, and the relevance of the smartphone features to biological dopaminergic deficit beyond that offered by the MDS-UPDRS-III. If confirmed, this combined clinical and digital framework could provide a cost-effective and widely accessible pre-screening tool for DaT imaging – bringing the potential for earlier intervention and more frequent monitoring into the hands of patients and clinicians alike.

## Methods

### Study design

The dataset used in this work is derived from a subset of participants taking part in the OPDC Discovery Cohort using a previously developed smartphone application^11,12,15^. The study involves human participants and was approved by the South Central – Oxford A Research Ethics Committee (IRAS number 188167). An additional set of RBD and PD participants from Genoa were included in the analysis, for which ethics approval was obtained from the local institutional board (CET Liguria - 184REG2017). There was no patient or public involvement in the design or conduct of this study.

During each study visit, participants underwent clinical and digital assessments (PD participants were assessed ON dopaminergic medication), imaging, and biological sampling. The protocol, including longitudinal clinical assessments, e.g MDS-UPDRS-III, performed as part of the Discovery cohort are detailed elsewhere and in the PD sample they were performed on existing medication^33,34^. Participants were excluded from the study if they had parkinsonism secondary to any other disorder than idiopathic PD or dementia preceding PD by one year. Controls were excluded on the basis of any known first- or second-degree family history of PD, history of stroke, alcohol, or drug abuse.

All participants in the Discovery cohort were given the opportunity to consent to digital smartphone motor assessments in clinic and/or at home using the OPDC smartphone app^11^. Smartphone assessment was performed in clinic, followed by at-home assessments over 1 week. Longitudinal assessments were performed at approximately 18-month intervals in willing participants. For the participants from the Genoa group, only in-clinic smartphone assessments were performed, at one time-point.

DaT brain scans were performed in a subgroup of willing participants, with numbers limited by overall funding available. Dopaminergic deficit was measured by 123I-ioflupane single photon emission computed tomography.

For the OPDC cohort, the DaT SPECT scan was performed at the Oxford University Hospitals NHS Foundation Trust, under the supervision of a consultant radiologist. Subjects were injected with 185 MBq +/-10\% of 123I-ioflupane (provided as DaTscanTM injection, GE Healthcare). Potassium iodide 120 mg was administered one hour prior to, and 24 h after, injection of 123I-ioflupane to block thyroid uptake. SPECT/CT images were acquired three hours post injection on a dual-headed gamma camera (Discovery 670 gamma camera, GE Healthcare, Haifa). SPECT parameters: 120 projections, 30 s per projection, 128 × 128 matrix. CT parameters: 16 slice, helical acquisition, 120 KV, 40 mA, noise index 30. The SPECT/CT data was reconstructed using HERMES Hybrid Recon (HERMES Medical Solutions, AB Stockholm) OSEM, 15 iterations, 4 subsets with attenuation correction from CT, collimator resolution recovery, and Monte Carlo scatter correction. The isotropic voxel size of reconstructed images was 2.21 mm^3^.

For the Genoa cohort, brain [^123^I]FP-CIT SPECT was acquired according to EANM guidelines^35^.

Data were acquired by means of a dual-headed Millennium VG camera (G.E. Healthcare). Acquisition started between 180 and 240 min after injection of [^123^I]FP-CIT and lasted 40 min. A “step-and-shoot” protocol was applied with a radius of rotation <15 cm, and 120 projections evenly spaced over 360° were generated. Total counts ranged between 2.0 and 2.5 million. The pixel size of the acquisition matrix was 2.4 mm, thanks to an electronic zoom (zoom factor ¼ 1.8) applied in the data collection phase. In the reconstruction phase, also a digital zoom was used and the resulting images were sampled by isotropic voxels with 2.33 mm sides. Projections were processed by means of the ordered subsets expectation maximisation (OSEM) algorithm (8 iterations, 10 subsets) followed by post filtering (3D Gaussian filter with full width-half maximum ¼ 8 mm). The OSEM algorithm included a proback pair accounting for collimator blur and photon attenuation. No compensation for scatter was performed. The 2Dþ1 approximation was applied in the simulation of the space-variant collimator blur, whereas photon attenuation was modelled with the approximation of a linear coefficient uniform inside the skull and equal to 0.11 cm^−1^.

The reconstructed [^123^I]FP-CIT SPECT images were processed using the BasGan software version 2 based on a high definition, 3D striatal template, derived from Talairach’s atlas^36^. Partial volume effect (PVE) correction is included in the process of uptake computation of caudate, putamen, and the occipital region background. The partial volume effect correction performed by the method consists of an activity assignment in a Talairach-Tornoux atlas-based 3-compartment model of basal ganglia. Background uptake was subtracted by putamen and caudate uptake as follows: (caudate or putamen uptake–background uptake)/background uptake, to generate specific to non-displaceable binding ratio (SBR) values. Partial volume correction, a feature included in the BasGan pipeline^36^, allows to reduce the impact of the limited SPECT spatial resolution of the assessment of midline structures.

### Data preprocessing

All smartphone data were collected from 2014 to 2024 using consumer-grade smartphones (Motorola, predominantly Motorola G model). Inclusion criteria: (1) all 7 tasks were completed, (2) the voice task was considered complete if the sustained phonation was ≥2 s long. The smartphone protocol is extensively detailed elsewhere^11^. Participants were asked to perform 7 short tasks (~8 min) to assess: (1) voice, (2) balance, (3) gait, (4) finger tapping, (5) reaction time, (6) rest tremor and (7) postural tremor, in order to emulate motor assessments commonly performed in the clinic by a trained clinician. The data were encrypted and timestamped. Smartphone assessments were included if they were within +/- one year of the DaT scan date.

The voice task in this work comprised of a sustained phonation of “aaah” (international phonetic alphabet /a:/), from which 339 features were extracted that quantify roughness in voice, monotonicity, variation in amplitude and frequency, etc. From the remaining 6 tasks, a total of 719 features were extracted, and for bimanual tasks, features were extracted from the dominant hand. For the reaction time task, features were extracted based on the time elapsed between stimulus (button on screen) and response (pressing/release of button). Spatial and temporal tapping task features were derived from the pixel coordinates and timing of the screen touch. For the accelerometer tasks, features were designed to quantify body motion. A comprehensive overview of the features has been reported in our previous study^11^, and an overview of the application is given in Supplementary Fig. 8.

DaT scans were annotated as normal or abnormal by a trained radiologist to the clinical diagnosis. A consensus panel of 3 imaging neuroscientists and co-authors (JK, MH and KG) reviewed the radiological report alongside the striatal binding ratio’s (SBRs) and Z-scores (uncorrected and corrected for age) using the BRASS (Hermes Medical Solutions) software and categorised each DaT scan as normal or abnormal^37^. BRASS software fits individual DaT SPECT data onto a template with pre-defined ROI, four striatal (left and right caudate and putamen) and two extra-striatal control regions. SBR and Z-scoring calculations are described below. This consensus diagnosis was used as the gold standard for the model.

The BRASS (Hermes Medical Solutions) calculations, as defined in the Hermes Medical Solutions BRASSTM Handbook version 6, are given as:

$$\mathrm{SBR}={\rm{R}}-1$$

$${\rm{UncorrectedZ}}-{\rm{score}}=\begin{array}{c}{\rm{Measured\; ratio}}+1-{\rm{Mean\; ratio}}\\ {\rm{SD\; ratio}}\end{array}$$

$$\mathrm{Age\; corrected}\,{\rm{Z}}-\mathrm{score}=\begin{array}{l}\mathrm{Measured\; ratio}-\mathrm{Mean\; ratio}+\mathrm{Age\; correction\; factor}\\ \mathrm{SD\; ratio}+1\end{array}$$

Where SBR is the Striatal (specific) binding ratio, R is the ratio (average counts in region/average counts in reference region). The Age correction factor is (Mean age - Measured age) × Slope*, where the mean age is 58 years (as per BRASS reference cohort). The reference region for DaT SPECT in BRASS is the cerebellum and will always be 1.

*Slopes 1 or 2 are used in this segmented regression model. Slope 1 if measured age < mean age and slope 2 if measured age > mean age.

### Models

For our classification task, we utilised both a logistic regression (LR) model and an XGBoost classifier to compare performance across linear and ensemble-based approaches. The classifiers were trained to classify normal versus abnormal, DaT scans across the cohort (PD, iRBD, and HC), using both in-clinic and at-home smartphone assessments when available. XGBoost is a commonly used machine learning model which has been shown to be competitive with other techniques^38^. The models were evaluated using 5-fold cross-validation (CV), stratified by participant. Thus, all recordings from a given participant were used either for training or testing, but not both, over a CV fold. To investigate the stability of the classifiers, the CV was also repeated (5x repeated 5-fold CV) across different splits. The smartphone feature set includes 1058 features in total. To identify the most salient features in the modelling, feature selection was performed using SHapley Additive exPlanations SHAP^17^ values. The models were evaluated with the top 10, 30, 50, 100, 300, and 500 features. The features were scaled to have a mean of 0 and unit variance, and missing values were imputed using the median of the in-sample data. We also report the performance of the model when adding clinical variables to the most salient feature set. “Clinical variable only” models were trained and evaluated using the same splits. Within each fold, 3-fold nested CV was used to optimise the hyperparameters of the XGBoost model. A random grid search was performed to determine the optimal number of estimators (number of trees), the learning rate, and the number of features used to build each tree. When using only one clinical variable to predict the output, one estimator was used.

The binary classification models were evaluated using the mean AUC over folds with the standard deviation. As there were multiple smartphone assessments matched to a given DaT scan label, the model output probabilities were averaged to give one classification for each DaT scan. Bootstrapping was conducted to calculate 95% confidence intervals (CIs) for the AUC values. The sensitivity and specificity were also calculated.

#### Predicting striatal DaT binding ratios

For each hemisphere, age-corrected binding ratios were predicted in four ROI: the right and left caudate and putamen. We then trained and evaluated both a simple decision tree regressor and an XGBoost regressor for each of these four ROIs, using both in-clinic and at-home assessments. As with the classifier, we averaged the outputs across all available smartphone assessments corresponding to each DaT scan. Model hyperparameters were optimised via a random grid search with 3-fold cross-validation, as previously described. We compared the performance of the smartphone-based model to both the naïve benchmark (mean in-sample prediction) and a clinical benchmark model using only the MDS-UPDRS-III. Regression performance was assessed using the root mean squared error (RMSE).

Descriptive statistics for groups: normal vs. abnormal, and pair-wise for HCs, RBD, and PD diagnostic groups with regards to the SBRs, were analysed. Significance for binary variables was given using the Mann–Whitney U-test. Two-sided Welch’s t-test was conducted for continuous variables. A one-way ANOVA with Tukey’s honestly significant difference (HSD) was conducted to test for differences between disease groups for each region. To assess differences in binding ratios across the four ROIs within each disease group, a repeated-measures ANOVA was performed, followed by pairwise (paired) t-tests with Bonferroni correction. All analysis was performed using Python version 3.10.4. Code is available through contacting the corresponding author.

## Supplementary information

Supplementary materials

## Data Availability

Access to the Oxford Parkinson’s Disease Centre (OPDC) dataset used in this study is available through a formal submission to the Data Access Committee who will review this and either support, decline, or request further information. See https://www.dpag.ox.ac.uk/opdc/research/external-collaborations for more information.

## Code availability

The model training and evaluation code can be made available by emailing the corresponding author of the manuscript.
