# Supplementary material for: Smartphone-based prediction of dopaminergic deficit in prodromal and manifest Parkinson’s disease
Source: NPJ Digit Med. 2025 Dec 1;8:783. doi: 10.1038/s41746-025-02148-2 (PMC12738540; doi:10.1038/s41746-025-02148-2)
Supplement: Supplementary file 1 — Supplementary materials [file 41746_2025_2148_MOESM1_ESM.pdf]

Smartphone-Based Prediction of Dopaminergic Deficit in Prodromal and Manifest Parkinson's Disease – Supplementary Materials

Supplementary Materials

Statistical significance of DaT striatal binding ratios

ROI	Comparison	Mean Diff.	95% CI Lower	95% CI Upper	p_adj	Significant
Right caudate	HC vs PD	-1.1123	-1.2353	-0.9894	<0.001	Yes
Right caudate	HC vs iRBD	-0.3169	-0.4335	-0.2002	<0.001	Yes
Right caudate	PD vs iRBD	0.7955	0.7182	0.8727	<0.001	Yes
Left caudate	HC vs PD	-0.9886	-1.1342	-0.8430	<0.001	Yes
Left caudate	HC vs iRBD	-0.3069	-0.4450	-0.1687	<0.001	Yes
Left caudate	PD vs iRBD	0.6818	0.5902	0.7733	<0.001	Yes
Right putamen	HC vs PD	-1.6372	-1.7545	-1.5198	<0.001	Yes
Right putamen	HC vs iRBD	-0.4300	-0.5413	-0.3186	<0.001	Yes
Right putamen	PD vs iRBD	1.2072	1.1334	1.2810	<0.001	Yes
Left putamen	HC vs PD	-1.5044	-1.6538	-1.3550	<0.001	Yes
Left putamen	HC vs iRBD	-0.3479	-0.4897	-0.2062	<0.001	Yes
Left putamen	PD vs iRBD	1.1564	1.0625	1.2504	<0.001	Yes

Supplementary Table 1. Between-Group Comparisons of DaT Binding Ratios by Region. One-way ANOVA followed by Tukey's HSD post hoc comparisons revealed significant differences indicated by p-values.

Diagnosis	ROI Comparison	t-Statistic	p_adj	Significant
**iRBD**	**Left caudate vs Left putamen**	**6.65**	**8.41E-08**	**Yes**

**iRBD**	Left caudate vs Right caudate	1.32	1.00	No
**iRBD**	**Left caudate vs Right putamen**	**7.53**	**3.09E-09**	**Yes**
**iRBD**	**Left putamen vs Right caudate**	**-5.07**	**2.89E-05**	**Yes**
**iRBD**	Left putamen vs Right putamen	1.53	0.792	No
**iRBD**	**Right caudate vs Right putamen**	**6.07**	**7.48E-07**	**Yes**
**PD**	**Left caudate vs Left putamen**	**10.98**	**1.41E-12**	**Yes**
**PD**	Left caudate vs Right caudate	-0.04	1.00	No
**PD**	**Left caudate vs Right putamen**	**7.74**	**1.51E-08**	**Yes**
**PD**	**Left putamen vs Right caudate**	**-9.68**	**5.04E-11**	**Yes**
**PD**	Left putamen vs Right putamen	1.05	1.00	No
**PD**	**Right caudate vs Right putamen**	**13.26**	**4.68E-15**	**Yes**
**HC**	Left caudate vs Left putamen	2.47	0.414	No
**HC**	Left caudate vs Right caudate	-0.93	1.00	No
**HC**	Left caudate vs Right putamen	1.32	1.00	No
**HC**	Left putamen vs Right caudate	-2.40	0.449	No
**HC**	Left putamen vs Right putamen	-0.15	1.00	No
**HC**	Right caudate vs Right putamen	1.83	0.844	No

Supplementary Table 2. Within-Group Comparisons of DaT Binding Ratios Across Brain Regions. Paired t-tests with Bonferroni correction were used to assess differences between regions within each diagnostic group. Significant differences in bold text.

For the sub-group analysis in supplementary Figure 3. the prediction probabilities were optimised on the training set using the grid of probability thresholds. They were optimsed overall, and not by subgroup.

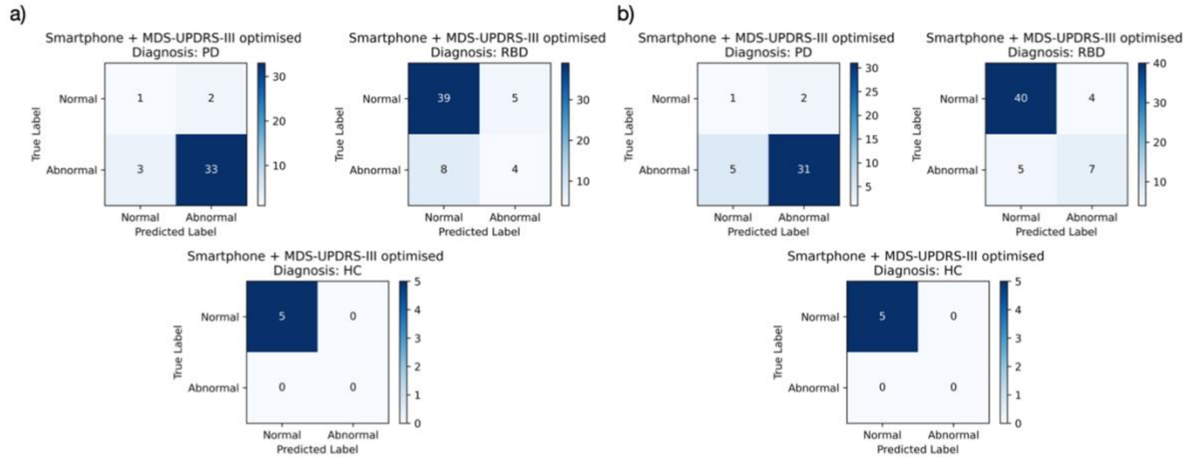

Supplementary Figure 1. Confusion matrices for the combined smartphone and MDS-UPDRS-III a) XGBoost and b) logistic regression models split by diagnosis group. PD: Parkinson’s disease. RBD: Rapid-eye-movement sleep behaviour disorder. HC: healthy controls.

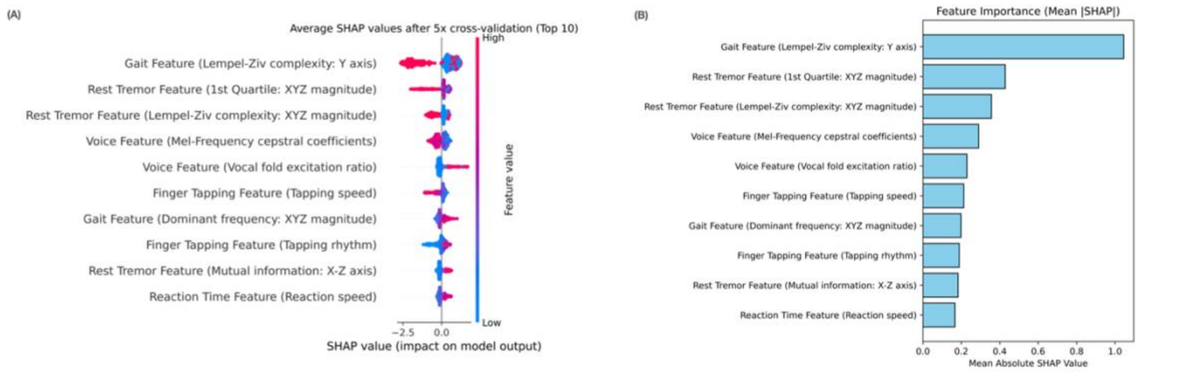

Supplementary Figure 2. Smartphone feature saliency (top 10 features). (A) Raw shap values for each observation, aggregated over 5 cross-validation folds. A negative SHAP value indicates contribution to lower probability of abnormal DaT scan. (B) Absolute SHAP values overall, aggregated across 5 cross-validation folds.

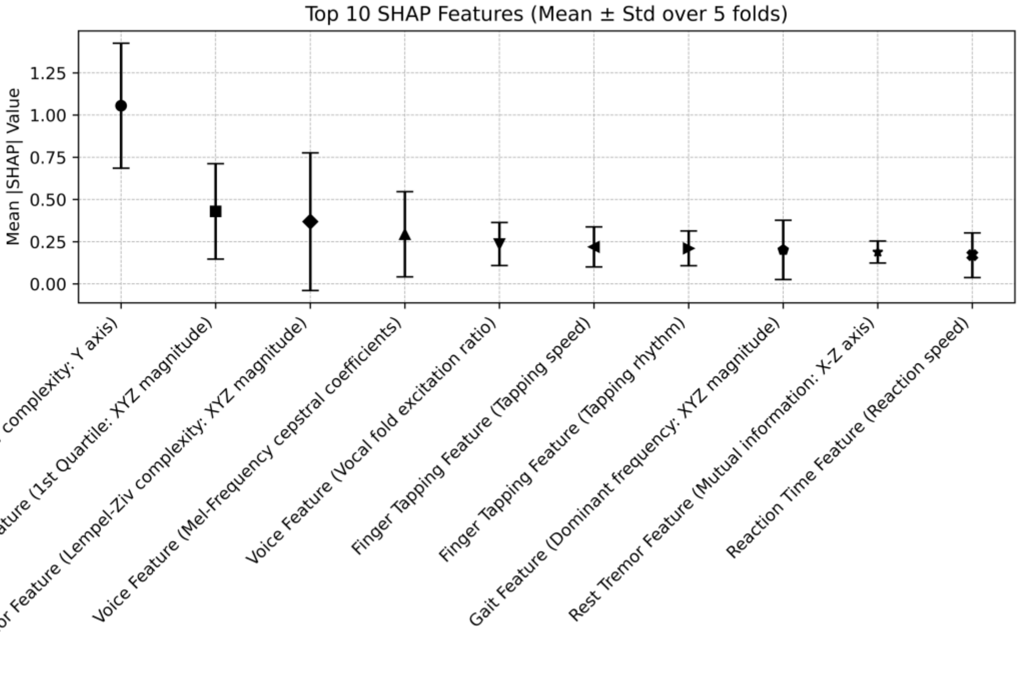

Supplementary Figure 3. Mean absolute SHAP values for the top 10 smartphone features with standard deviation across the 5 cross-validation folds.

Repeat	Accuracy (Mean ± Std)	Precision (Mean ± Std)	Recall (Mean ± Std)	AUC (Mean ± Std)	Sensitivity (Mean ± Std)	Specificity (Mean ± Std)
**1**	0.83 ± 0.06	0.84 ± 0.07	0.82 ± 0.05	0.86 ± 0.03	0.75 ± 0.07	0.89 ± 0.06
**2**	0.83 ± 0.05	0.83 ± 0.06	0.83 ± 0.05	0.87 ± 0.06	0.76 ± 0.10	0.90 ± 0.06
**3**	0.82 ± 0.06	0.82 ± 0.06	0.82 ± 0.06	0.87 ± 0.06	0.74 ± 0.10	0.90 ± 0.08
**4**	0.81 ± 0.07	0.82 ± 0.07	0.81 ± 0.07	0.86 ± 0.06	0.73 ± 0.13	0.89 ± 0.08
**5**	0.81 ± 0.07	0.82 ± 0.07	0.81 ± 0.08	0.87 ± 0.07	0.74 ± 0.14	0.88 ± 0.10

Supplementary Table 3. Mean performance metrics with standard deviation (Std) across 5 repeats of 5-fold cross validation using the XGBoost model (MDS-UPDRS-III + smartphone features) to predict normal vs abnormal DaT scans.

Repeat	Accuracy (Mean ± Std)	Precision (Mean ± Std)	Recall (Mean ± Std)	AUC (Mean ± Std)	Sensitivity (Mean ± Std)	Specificity (Mean ± Std)
**1**	0.80 ± 0.08	0.80 ± 0.07	0.80 ± 0.07	0.84 ± 0.05	0.74 ± 0.11	0.85 ± 0.08

**2**	0.82 ± 0.07	0.81 ± 0.06	0.83 ± 0.07	0.87 ± 0.06	0.78 ± 0.12	0.87 ± 0.08
**3**	0.82 ± 0.07	0.82 ± 0.07	0.83 ± 0.08	0.87 ± 0.07	0.76 ± 0.14	0.89 ± 0.09
**4**	0.81 ± 0.08	0.82 ± 0.09	0.81 ± 0.09	0.86 ± 0.08	0.75 ± 0.15	0.88 ± 0.10
**5**	0.82 ± 0.08	0.82 ± 0.08	0.82 ± 0.08	0.87 ± 0.08	0.76 ± 0.14	0.88 ± 0.10

Supplementary Table 4. Mean performance metrics with standard deviation (Std) across 5 repeats of 5-fold cross validation using the logistic regression (MDS-UPDRS-III + smartphone features) model to predict normal vs abnormal DaT scans.

**Variable**	**Coefficient**	**Std error**	**P-value**
Intercept	0.18	0.01	<0.00001
True label	0.43	0.02	<0.00001
Sex	0.16	0.02	<0.00001
Age (standardised)	-0.01	0.01	0.22

Supplementary Table 5. Multiple linear regression. Applied to the probability output from the XGboost model using both smartphone features and the MDS-UPDRS-III as input features. Significance indicated by p-values.

Predicting striatal binding ratios

**Model**	**Right Putamen**	**Left Putamen**	**Right Caudate**	**Left Caudate**
**DT (feature selection)**	RMSE: 0.75 +/- 0.45<br>(50 features)	RMSE: 0.68 +/- 0.39<br>(50 features)	RMSE: 0.60 +/- 0.37<br>(200 features)	RMSE: 0.66 +/- 0.44<br>(200 features)
**MDS-UPDRS-III**	RMSE: 0.56 +/- 0.36	**RMSE: 0.58 +/- 0.32**	RMSE: 0.53 +/- 0.32	RMSE: 0.60 +/- 0.37

**DT MDS-UPDRS-III + Smartphone top features**	RMSE: 0.64 +/- 0.41	RMSE: 0.61 +/- 0.34	RMSE: 0.61 +/- 0.38	RMSE: 0.65 +/- 0.37
**Combination prediction (mean)**	**RMSE: 0.56 +/- 0.33**	RMSE: 0.59 +/- 0.32	**RMSE: 0.50 +/- 0.30**	**RMSE: 0.57 +/- 0.35**

Supplementary Table 6. Regression results for predicting the DaT ratios corresponding to the four regions of interest using decision tree (DT) models. RMSE: root mean squared error, presented as mean ± standard deviation. Note: lower RMSE values are better. The best models are highlighted in bold.

**Model combination (Smartphone + clinical)**	**Right Putamen – residuals from clinical model**
**Naive benchmark + DT (Mean in-sample prediction)**	RMSE: 0.56 + /- 0.36
**XGboost + DT**	RMSE: 0.50 + /- 0.32
**XGboost + DT Error sample weights**	RMSE: 0.52 + /- 0.32

Supplementary Table 7. Regression results for predicting the residuals from the clinical MDS-UPDRS-III only model in predicting the right putamen using a naïve benchmarch and an XGBoost model with the smartphone features as input. Error sample weights indicate that the magnitude of the residuals were used as sample weights for the smartphone XGBoost model when predicting the clinical model residuals.

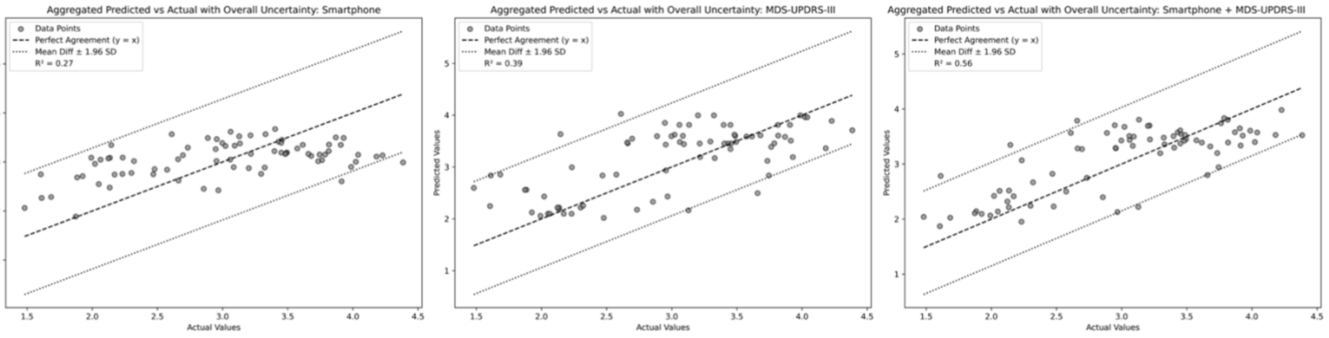

Supplementary Figure 4. Predicted versus actual binding ratios under three modeling approaches—(left) smartphone-only features, (middle) MDS-UPDRS-III–only, and (right) combined smartphone + MDS-UPDRS-III—each with overall uncertainty bounds. Points represent aggregated predictions for individual scans, and dashed lines denote estimated error margins.

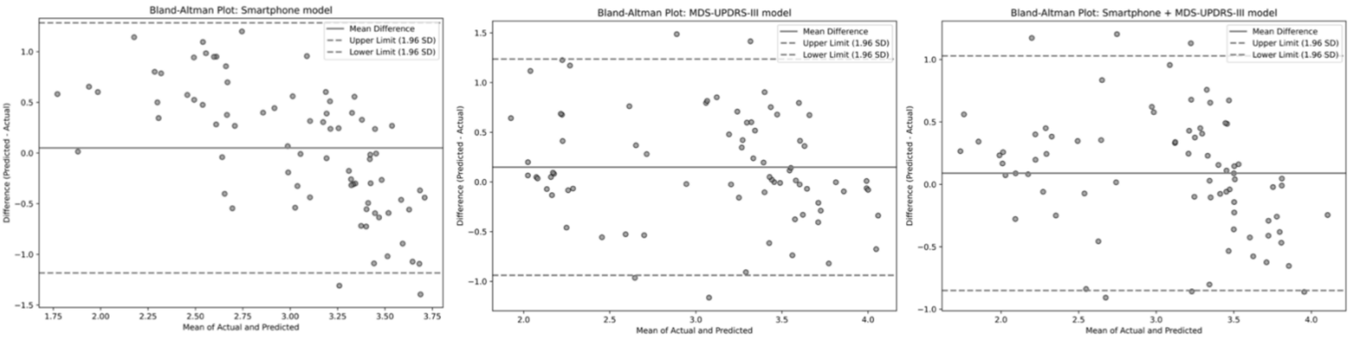

Supplementary Figure 5. Bland-Altman plots comparing actual and predicted binding ratios under three modeling approaches—(left) smartphone-only, (middle) MDS-UPDRS-III–only, and (right) combined smartphone + MDS-UPDRS-III. The solid line indicates the mean difference, and dashed lines represent the 95% limits of agreement.

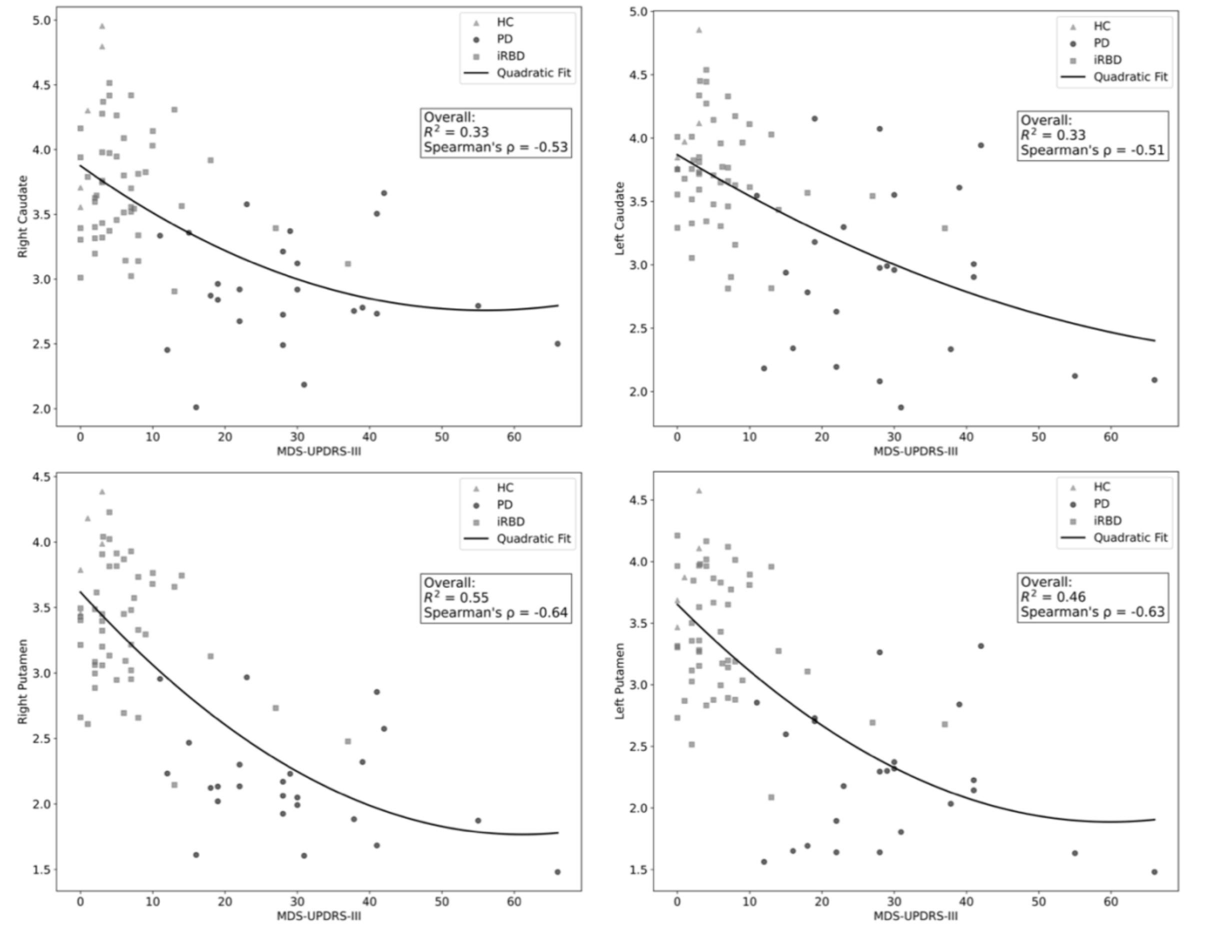

Supplementary Figure 6. Quadratic fits illustrating the relationship between MDS-UPDRS-III scores and DaT binding ratios in the right/left caudate (top row) and right/left putamen (bottom row). Points represent healthy controls (HC), individuals with iRBD, and patients with PD. Each subplot shows the overall coefficient of determination (R^2) and Spearman's correlation (ρ) across all participants.

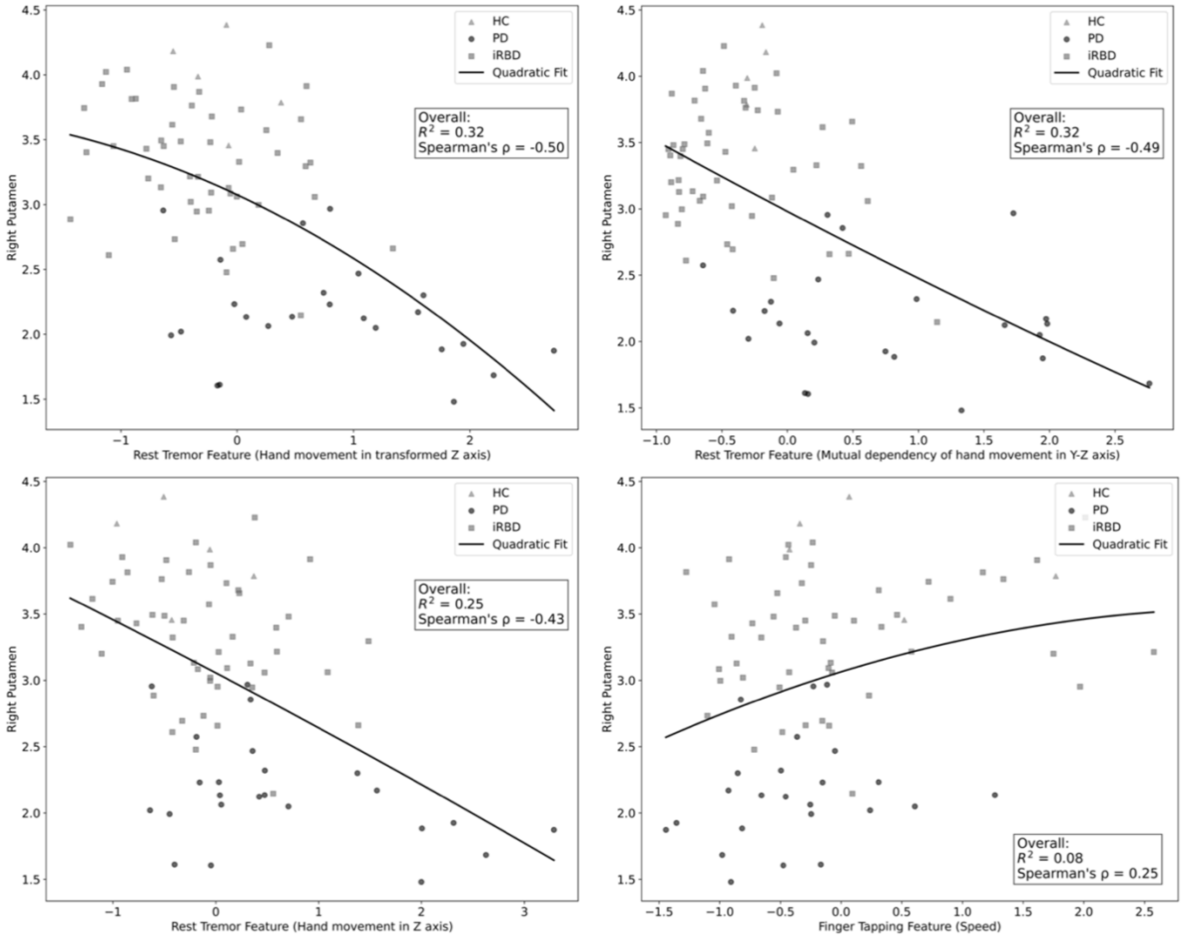

Supplementary Figure 7. Quadratic fits illustrating the relationship between four of the top 10 smartphone features and DaT binding ratios in the right putamen. Points represent healthy controls (HC), individuals with IRBD, and patients with PD. Each subplot shows the overall coefficient of determination (R^2) and Spearman correlation's (ρ) across all participants.

The OPDC Smartphone Application

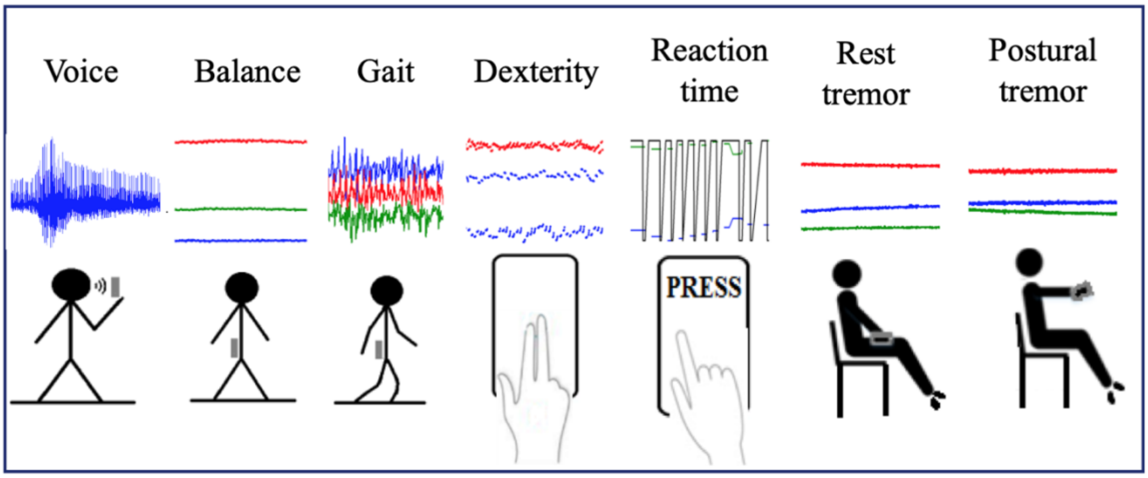

Supplementary Figure 8. Overview of the Oxford Parkinson's Disease Centre (OPDC) smartphone application motor assessment.

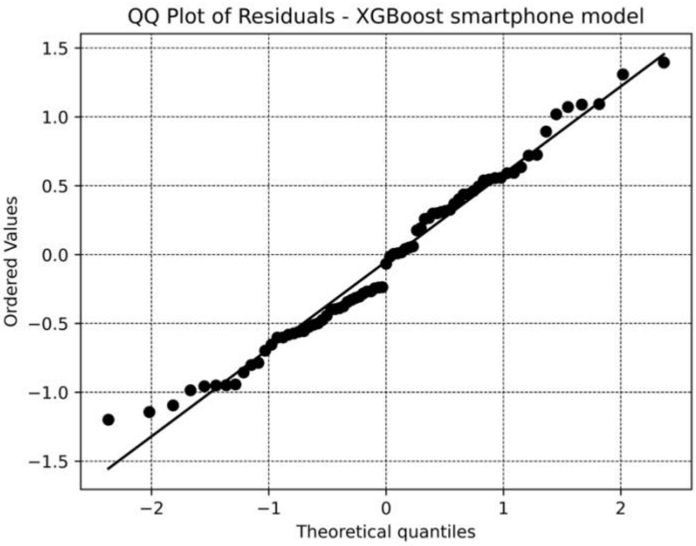

Supplementary Figure 9. Quantile-Quantile (Q-Q) of the predictions of the right putamen striatal binding ratio using the smartphone XGBoost model.

Given that the tapping task could be participant to practice effects, the significance of any practice effect in the top tapping feature (tapping frequency) was examined. The tapping feature value from the first smartphone assessment was compared to feature values from the subsequent assessments within a two-week period. Assessments were included if at least 5 smartphone assessments were completed within this period from the date of the first assessment. The change between the feature value of the first assessment and the mean feature value across the following two weeks was insignificant (p-value = 0.26), as determined by a T-test.

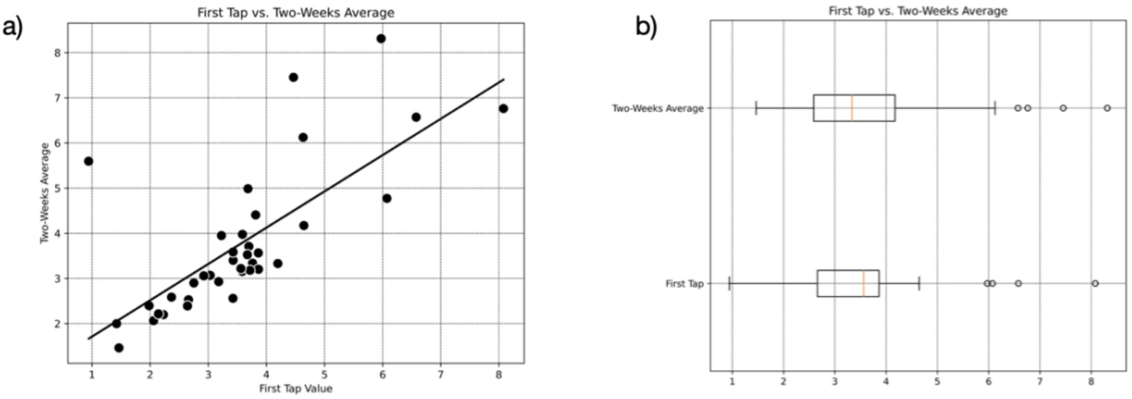

Supplementary Figure 10. Comparison of the most salient tapping feature (tapping speed) in a subset of the patients using the first smartphone recording and all subsequent recordings during a two-week period. 14 PD, 18 RBD, and 5 Control participants. T-test p-value = 0.26.
